# Potential factors of cytokeratin fragment 21-1 and cancer embryonic antigen for mediastinal lymph node metastasis in lung cancer

**DOI:** 10.3389/fgene.2022.1009141

**Published:** 2022-09-13

**Authors:** Jing Tang, Hui-Ye Shu, Tie Sun, Li-Juan Zhang, Min Kang, Ping Ying, Qian Ling, Jie Zou, Xu-Lin Liao, Yi-Xin Wang, Hong Wei, Yi Shao

**Affiliations:** ^1^ Department of Oncology, The Affiliated Zhuzhou Hospital Xiangya Medical College, Central South University, Zhuzhou, Hunan, China; ^2^ Department of Ophthalmology, Jiangxi Branch of National Clinical Research Center for Ocular Disease, The First Affiliated Hospital of Nanchang University, Nanchang, Jiangxi, China; ^3^ Department of Ophthalmology and Visual Sciences, The Chinese University of Hong Kong, Hong Kong, China; ^4^ School of Optometry and Vision Science, Cardiff University, Cardiff, United Kingdom

**Keywords:** lung cancer, mediastinal lymph node metastasis potential indicators, tumor blood markers, cancer, risk factors

## Abstract

**Objective:** Lung cancer is a common malignant tumor, characterized by being difficult to detect and lacking specific clinical manifestations. This study aimed to find out the risk factors of mediastinal lymph node metastasis and explore the correlation between serum tumor markers and mediastinal lymph node metastasis and lung cancer prognosis.

**Methods:** A retrospective study of 3,042 lung cancer patients (330 patients with mediastinal lymph node metastasis and 2,712 patients without mediastinal lymph node metastasis) collected from the First Affiliated Hospital of Nanchang University from April 1999 to July 2020. The patients were divided into two groups, namely, mediastinal lymph node metastasis group and non-mediastinal lymph node metastasis group. Student’s t test, non-parametric rank sum test and chi-square test were used to describe whether there is a significant difference between the two groups. We compared the serum biomarkers of the two groups of patients, including exploring serum alkaline phosphatase (ALP), calcium hemoglobin (HB), alpha-fetoprotein (AFP), carcinoembryonic antigen (CEA), CA125, CA-199, CA -153, cytokeratin fragment 19 (CYFRA 21-1), total prostate specific antigen (TPSA), neuron-specific enolase (NSE) levels and the incidence and prognosis of lung cancer mediastinal lymph node metastasis. Binary logistic regression analysis was used to determine its risk factors, and receiver operating curve (ROC) analysis was used to evaluate its diagnostic value for mediastinal lymph node metastasis.

**Results:** Binary logistic regression analysis showed that carcinoembryonic antigen and CYFRA 21-1 were independent risk factors for mediastinal lymph node metastasis in patients with lung cancer (*p* < 0.001 and *p* = 0.002, respectively). The sensitivity and specificity of CEA for the diagnosis of mediastinal lymph node metastasis were 90.2 and 7.6%, respectively; CYFRA 21-1 were 0.6 and 99.0%, respectively.

**Conclusion:** Serum CEA and CYFRA 21-1 have predictive value in the diagnosis of mediastinal lymph node metastasis in patients with lung cancer.

## Introduction

The morbidity and mortality of lung cancer remain high, and the prognosis of lung cancer patients has not been effectively improved. The preferred treatment for lung cancer is surgery. Postoperative radiotherapy, chemotherapy, biological immunotherapy, and the advent of targeted drugs have all led to increases in the survival time of patients. Lymph node metastasis is one of the main metastatic pathways of lung cancer and is an important determinant of lung cancer stage. Lung cancer has no specific symptoms in the early stages. The cancer cells pass through the bronchus and the lymphatic vessels around the pulmonary blood vessels. They first invade the adjacent lung or lymph nodes around the bronchi and then reach the hilar or subcarinal lymph nodes. They may then spread to the mediastinum and paratracheal lymph nodes, and further to the clavicular or cervical lymph nodes. Immune checkpoint inhibitors is a currently widely used tumor treatment method. Through the development of various related clinical trials, immune checkpoint inhibitors have made many breakthroughs in the efficacy, prognosis and disease prediction of lung cancer (Jain et al., 2018; Bai et al., 2020).

Tumor marker detection has become one a routine detection method, but there is still no ideal marker for clinical use as an indicator of lung cancer metastasis and prognosis. Single marker detection systems often suffer from low specificity (Weinberger et al., 2002). The same tumor can contain a variety of tumor markers. Different tissue types of the same tumor can express the same tumor markers or different tumor markers, so combined detection of multiple tumor markers can improve the diagnostic sensitivity of cancer.

A number of studies have identified distinct factors that are associated with lymph node metastasis in lung cancer. Specific substances detected in the lymph nodes or blood can predicts tumor. In this study, the medical records of lung cancer patients from the First Affiliated Hospital of Nanchang University were collected. Based on serological examination of a large number of lung cancer patients, we screened patients with lung cancer mediastinal lymph node metastasis and analyzed their tumor marker content to identify risk factors. We aimed to establish a standard by which to distinguish between mediastinal lymph node metastasis and non-mediastinal lymph node metastasis and to facilitate the development of further targeted anticancer treatment strategies for lung cancer patients.

## Materials and methods

### Ethics statement and study design

All patients in this study volunteered to participate, and the study was approved by the Medical Research Ethics Committee of the First Affiliated Hospital of Nanchang University. In this study, patients diagnosed with lung cancer between September 1999 and July 2020 were selected. Patients with mediastinal lymph node metastasis were screened, and their medical records and serological data were compared with those of patients without mediastinal lymph node metastasis. A pathological section obtained by surgical resection or biopsy was used to accurately diagnose the lung cancer of the patient. Computerized tomography (CT) and magnetic resonance imaging (MRI) were used to diagnose mediastinal lymph node metastasis in lung cancer, and data on serum tumor markers were recorded. Patients with primary mediastinal malignancies, benign mediastinal tumors, and secondary mediastinal cancer were excluded. The inclusion criteria for the without mediastinal lymph node metastasis group were patients without organ metastases.

### Data collection

We collected various clinical data, including age, gender, time of diagnosis, lesion metastasis, and treatment, from medical records of patients with mediastinal lymph node metastasis and analyzed serum tumor markers, including alkaline phosphatase, serum calcium, HB, alpha-fetoprotein (AFP), carcinoembryonic antigen (CEA), neuron-specific enolase (NSE), cytokeratin fragment 19 (CYFRA 21-1), CA-125, CA-153, CA-199, and free prostate-specific antigen (FPSA).

### Statistical analyses

We analyzed the differences between tumor markers in the mediastinal lymph node metastasis group and the non-mediastinal lymph node metastasis group by an independent *t*-test. A binary logistic regression model was then applied to identify independent risk factors for mediastinal lymph node metastasis. A receiver operating curve (ROC) curve was generated, and the area under the curve (AUC) was calculated. Then, we used Microsoft Excel 2010 software (Microsoft corporation,United States) to calculate the cut-off value, sensitivity, and specificity of risk factors. All statistical analyses were performed using SPSS 20.0 (SPSS, IBM, United States) and Excel 2010 software. *p* values < 0.05 indicates statistical significance.

## Results

### Demographics and clinical characteristics

**FIGURE 1:**
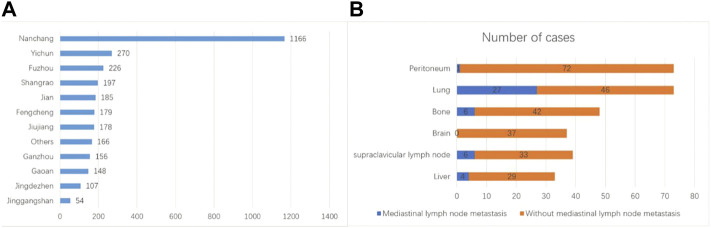
Clinical features of the patients. **(A)** Geographic location of the patients. **(B)** Anatomical locations of metastasis and corresponding incidences.

**FIGURE 2:**
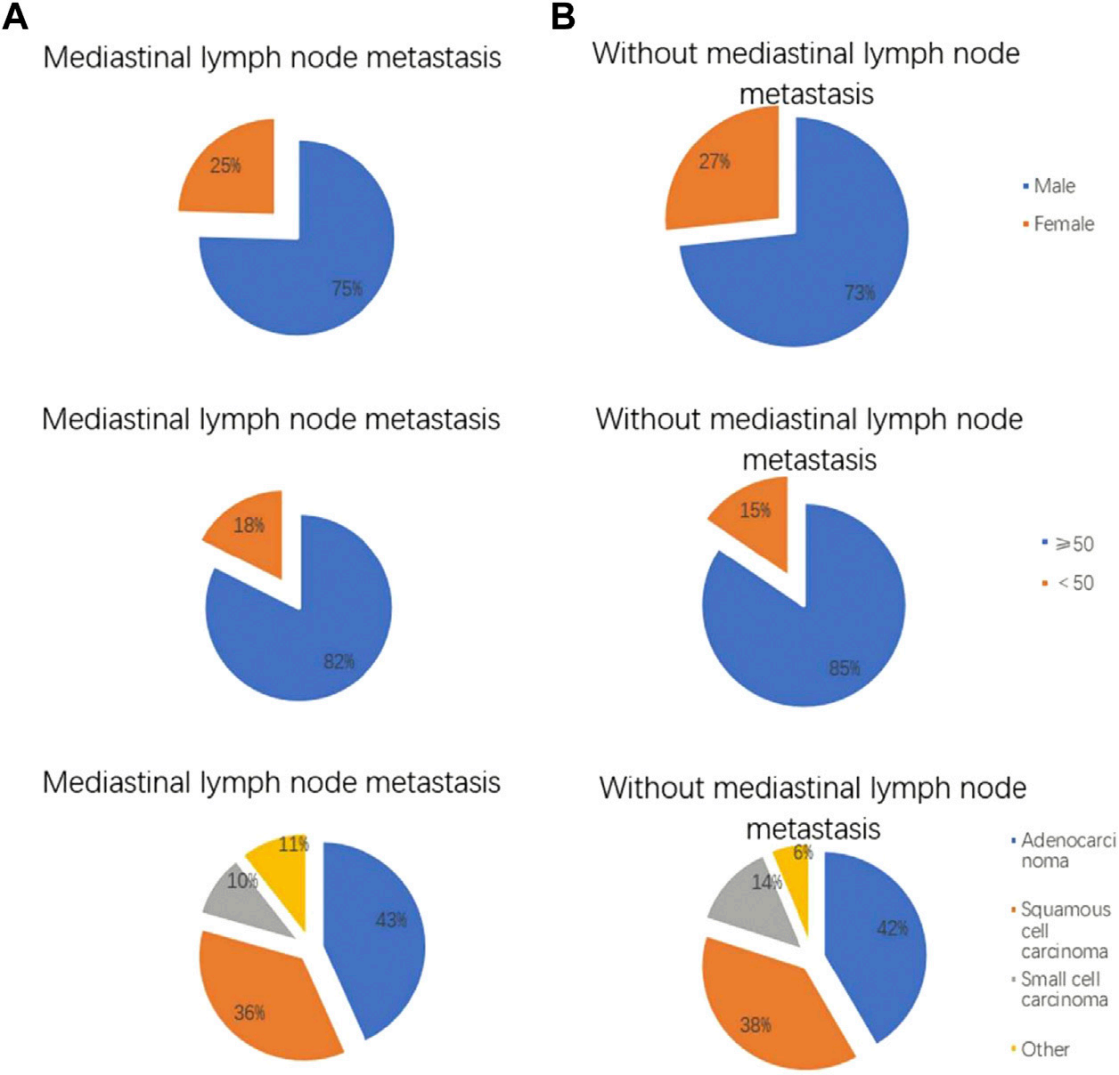
Clinical features of lung cancer patients with and without mediastinal lymph node metastasis. The **(A)** shows lung cancer patients with mediastinal lymph node metastasis, and the **(B)** shows lung cancer patients without mediastinal lymph node metastasis. Gender distribution of lung cancer patients with and without mediastinal lymph node metastasis. Age distribution of lung cancer patients with and without mediastinal lymph node metastasis. Pathological features of lung cancer patients with and without mediastinal lymph node metastasis.

**FIGURE 3:**
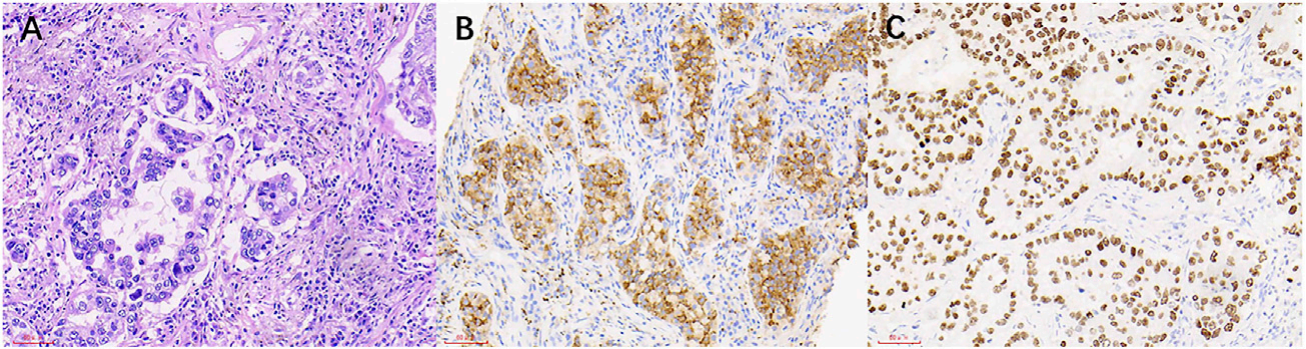
The HE staining and IHC images from lung cancer patients with mediastinal lymph node metastasis. **(A)** Lung cancer (HE × 200). **(B)** NapsinA (+) (SP × 200). **(C)** TTF-1 (+) (SP × 200).

In this study, 330 cases of lung cancer with mediastinal lymph node metastasis and 2,712 cases of lung cancers without mediastinal lymph node metastasis were collected. The mean ages of lung cancer patients with and without mediastinal lymph node metastasis were 59.6 ± 10.5 and 60.3 ± 10.8 years, respectively. According to the chi-squared test and Student’s t-test, there were no significant differences in gender or age between the lung cancer groups with and without mediastinal lymph node metastasis (*p* > 0.05). In contrast, significant differences were noted for the different histopathological types between the two groups (*p* < 0.05). There was a statistically significant difference in the pathological type between the mediastinal lymph node metastasis group and lung cancers without mediastinal lymph node metastasis (*p* = 0.012). Furthermore, the incidence of adenocarcinoma was the highest among the different histopathological types. Most patients had been treated with chemotherapy since the onset of the disease. Detailed clinical data of all patients involved in the study are provided in Table 1 and Figures 1–3.

**TABLE 1:** The clinical characteristics of patients with lung cancer.

Patient characteristics	Group with mediastinal lymph node metastasis (%)	Group without mediastinal lymph node metastasis (%)	*p* value^d^
	(*n* = 330)	(*n* = 2,712)	
Gender^a^			
Male	249 (75.5)	1988 (73.3)	0.485
Female	81 (24.5)	724 (26.7)	
Age^b^			
Mean	59.6 ± 10.5	60.3 ± 10.8	0.553
Histopathological type^c^			
Adenocarcinoma	143 (45.2)	1,128 (41.6)	0.012
Squamous cell carcinoma	119 (34.2)	1,041 (38.4)	
Small cell carcinoma	33 (10.0)	370 (13.6)	
Other	35 (10.6)	173 (6.4)	

a Chi-squared test.

b Student’s t-test.

c Comparison between the lung cancer group with brain metastasis and the lung cancer group without brain metastasis.

d *p* value < 0.05 was considered statistically significant.

### Clinical data and risk factors of mediastinal lymph node metastasis in lung cancer

After comparing data on tumor biomarkers in lung cancer patients with and without mediastinal lymph node metastasis, we found that the concentrations of AFP, CEA, and CYFRA 21-1 were significantly higher in patients with mediastinal lymph node metastasis, while HB was higher in patients without mediastinal lymph node metastasis (*p* < 0.05) (Table 2). There were no significant differences in serum ALP, calcium, CA-125, CA-199, CA-153, TPSA, NSE, between the two groups (*p* > 0.05) (Table 2). Based on binary logistic regression, CEA and CYFRA 21-1 appear to be independent risk factors for mediastinal lymph node metastasis in lung cancer. More detailed results are shown in Table 3.

**TABLE 2:** Differences in tumor biomarkers between lung cancer patients with and without mediastinal lymph node metastasis.

Tumor biomarkers	Mediastinal lymph node metastasis group	Without mediastinal lymph node metastasis group	t	*p* value
ALP (U/L)	92.48 ± 100.03	93.18 ± 77.32	0.159	0.8736
Calcium (nmol/L)	2.20 ± 0.22	2.29 ± 15.29	0.624	0.5325
AFP (ng/ml)	2.16 ± 1.04	1.75 ± 1.61	4.981	<0.001
CEA (ng/ml)	69.12 ± 340.77	44.25 ± 231.54	1.680	<0.001
CA-125 (U/ml)	76.03 ± 163.6	71.44 ± 191.25	0.396	0.6915
CA-199 (U/ml)	46.72 ± 199.1	46.28 ± 439.44	0.005	0.9960
CA-153 (U/ml)	21.96 ± 33.6	20.53 ± 34.54	0.694	0.4875
CYFRA 21-1 (ng/ml)	11.3 ± 26.55	9.56 ± 30.42	1.01	0.003
TPSA (ng/L)	2.02 ± 4.37	1.63 ± 3.73	1.71	0.0871
NSE (μg/L)	26.81 ± 41.57	26.12 ± 42.89	0.258	0.7963
HB (g/L)	115.75 ± 19.18	119.44 ± 19.15	3.10	0.0019

Notes: Apply *t*-test analysis. *p* < 0.05 indicated statistically significant differences. Abbreviations: ALP, alkaline phosphatase; HB, calcium hemoglobin; AFP, alpha fetoprotein; CEA,Cancer embryonic antigen; CYFRA 21-1, cytokeratin fragment 19; TPSA, total prostate specific antigen ;NSE, and neuron-specific enolase.

**TABLE 3:** Risk factors in lung cancer patients with mediastinal lymph node metastasis.

Factors	B	Exp(B)	OR (95% CI)	*p* value
AFP	0.127	1.135	1.059–1.216	0.134
CEA	0.000	1.000	1.000–1.001	<0.001
CYFRA 21-1	0.001	1.001	0.998–1.004	0.002
HB (g/L)	−0.009	0.991	0.985–0.997	0.068

Notes: Binary logistic regression analysis was applied. *p* < 0.05 indicated statistically significant differences. Abbreviations: HB, calcium hemoglobin; AFP, alpha fetoprotein; CEA, Cancer embryonic antigen; CYFRA 21-1, cytokeratin fragment 19.

### Cut-off value, sensitivity, and specificity of CEA and CYFRA 21-1 for the diagnosis of mediastinal lymph node metastasis in lung cancer

**FIGURE 4:**
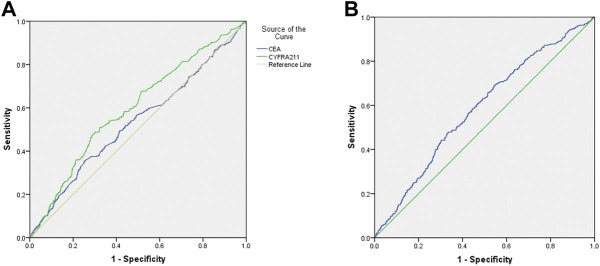
**(A)**ROC curve of CEA and CYFRA 21-1 levels in lung cancer patients with mediastinal lymph node metastasis. **(B)**ROC curve of CEA + CYFRA 21-1 in lung cancer patients with mediastinal lymph node metastasis.

Table 4 shows that the cut-off values for CEA and CYFRA 21-1 are 1.005 ng/ml and 135.31 ng/ml, respectively, and the area under the curve (AUC) of CYFRA 21-1 is the highest. The sensitivity and specificity for the diagnosis of mediastinal lymph node metastasis were CEA, 90.2 and 7.6%, respectively; CYFRA 21-1 was 0.6 and 99.0%, respectively. Figure 4A shows the receiver operating curve (ROC) curves for CEA and CYFRA 21-1, each as a single factor. We then tested the combination of these two risk factors in pairs, and Figure 4B shows the ROC curve for the CEA + CYFRA 21-1 combination. We found that the combination of CEA + CYFRA 21-1 has a higher AUC value of 0.585. The sensitivity and specificity of CEA + CYFRA 21-1 are shown in Table 4, and results were statistically significant (*p* < 0.05).

**TABLE 4:** Critical value, sensitivity, specificity and AUC of CEA and CYFRA 21-1 in lung cancer patients with mediastinal lymph node metastasis.

Factor	Cut-off value	Sensitivity (%)	Specificity (%)	AUC	*p* value
CYFRA 21-1 (ng/ml)	135.31	0.6	99.0	0.596	0.002
CEA (ng/ml)	1.005	90.2	7.6	0.533	<0.001
CEA + CYFRA 21-1	1,228.6	0.3	99.6	0.585	<0.001

Notes: Sensitivity and specificity were obtained at the cut-off value. *p* < 0.05 indicates statistically significant differences. Abbreviations: CEA, Cancer embryonic antigen; CYFRA 21-1, cytokeratin fragment 19.

## Discussion

Studies have shown that lymphatic metastasis occurs in papillary thyroid cancer. (Wang et al., 2019; Wen et al., 2019). Another scholar found that (Shimazu et al., 2019) used a one-step nucleic acid amplification test to detect lymph node metastasis of breast cancer, and in our study, serological tests were also found to predict lymph node metastasis of lung cancer. Studies have shown that (Han et al., 2019) lung cancer is prone to various types of metastasis, such as non-small cell lung cancer will occur bone metastasis.

Marchi et al. (Marchi et al., 2008). Used proteomics technology to screen the serum markers of lung cancer brain metastasis and found that patients with lung cancer brain metastasis had higher levels of ProApolipoprotein A1 and S100beta than those with lung cancer and cerebral ischemia. Roberts et al. (Roberts et al., 2002) first discovered the relationship between abnormal expression of retinol binding protein (RBP) and malignant metastasis of ovarian cancer epithelial cells, and speculated that due to the down-regulation and loss of RBP expression, it destroyed the metabolism of retinol and the production of retinoic acid. Promotes gene damage, leading to malignant transformation of ovarian epidermal cells.

At present, most clinicians’ method of clinical staging of lung cancer is a multidisciplinary diagnosis method based on chest CT scan (Liam et al., 2015), but chest CT is not specific for the diagnosis of intrapulmonary lymph node metastasis of lung cancer, and accuracy is not accurate. Cannot be used as a basis for surgical clearance (Zhang et al., 2019). Xu et al. (Xu et al., 2003) reported that the sensitivity, specificity and accuracy of PET in the diagnosis of mediastinal lymph node metastasis were 100, 93 and 94%, respectively, and the number and location of positive lymph nodes were completely consistent with pathological results.

The relationship between tumor size and invasion and lymph node metastasis is currently reported. Li Yu et al. (Li et al., 2000) summarized 386 cases of pathological data and thought that with the increase of tumor and increased invasion, the chance of lymph node metastasis increased significantly. Min Kong et al. (Kong et al., 2017) analyzed 1,156 patients, and the test results showed that there is some correlation between lymph node metastasis and the size of the primary tumor.

The intrathoracic lymphatic drainage route of lung cancer is usually performed according to a certain rule, that is, from the near to the far side, from the top to the bottom, from the lung to the mediastinum to the mediastinum, (Ndiaye et al., 2016), regardless of the location and severity, most cases are one station. In some cases, the transfer order of each station can change, or even jump. This situation has been reported in the literature at home and abroad. (Yoshino et al., 1996; Celikoglu et al., 2010).

According to the original location of the tumor, Watanabe et al. (Watanabe et al., 1990) divided the mediastinal lymph nodes into upper and lower parts according to the tracheal bifurcation. The incidence of lung cancer in the lower lobe was 22%, and the incidence of lung cancer in the upper lobe was 8%. Advocate extensive mediastinal lymph node dissection. However, extensive lymph node dissection can directly affect the patient’s survival. Funatsu et al. (Funatsu et al., 1994) found that the difference in survival rate may be related to the low immunity caused by mediastinal lymph node dissection. Additionally, the higher the Topography; Lymph Node; Metastasis (TNM)stage, the higher the serum CEA level. Some studies have found that the preoperative CEA level is related to non-small cell carcinoma survival. There have reported important factors related to independence (Okada et al., 2003; Yu et al., 2014).

CYFRA 21-1 is currently considered to be the main tumor marker used for the diagnosis of lung cancer. It is primarily distributed throughout the cytoplasm of the stratified tumor epithelium. When a cell dies, CYFRA 21-1 is released into the blood as a lysed fragment, resulting in an increase in serum levels. Pujol et al. reported that CYFRA 21-1 is an independent prognostic factor in lung cancer (Pujol et al., 2004).

In our study, we collected serum and assessed ALP, calcium, HB, AFP, CEA, CA-125, CA-199, CA-153, CYFRA 21-1, TPSA, and NSE levels. Relative to lung cancer patients without mediastinal lymph node metastasis, the concentrations of AFP, CEA, and CYFRA 21-1 in lung cancer patients with mediastinal lymph node metastasis were found to be extremely high, while HB was found to be lower (*p* < 0.05). Based on previous studies, we chose CEA and CYFRA 21-1 as independent risk factors for lung cancer patients with mediastinal lymph node metastasis (*p* < 0.01 and *p* = 0.002, respectively). Furthermore, we assessed the cut-off, sensitivity, specificity, and AUC of CEA and CYFRA 21-1 levels. Finally, we conclude that CEA, and CYFRA 21-1 are risk factors for mediastinal lymph node metastasis in lung cancer.

By using the final ROC curve of these serum biomarkers to provide reliable clinical indicators, we can conclude that CEA and CYFRA 21-1 have cut-off values of 1.005 ng/ml and 135.31 ng/ml, respectively, in lung cancer patients with mediastinal lymph node metastasis. CYFRA 21-1 had the highest AUC, demonstrating that it had the highest accuracy in distinguishing between lung cancer patients with and without mediastinal lymph node metastasis. On this basis, we utilized further detailed diagnostic techniques to diagnose or rule out mediastinal lymph node metastasis without providing a basis for follow-up treatment. Unlike previous studies, this study showed that the combination of CEA + CYFRA 21-1 had a higher AUC value of 0.585. Therefore, we believe that the combination of CEA + CYFRA 21-1 can also be used as a predictor of mediastinal lymph node metastasis in lung cancer (the higher the level of CEA + CYFRA 21-1, the greater the likelihood of mediastinal lymph node metastasis in lung cancer patients). We also summarized the risk factors for lung cancer metastasis in previous studies (Table 5).

**TABLE 5:** The risk factors of metastases of lung cancer.

Author	Year	Histopathological type	Metastatic sites	Risk factor
Morita et al. (Morita et al., 2019)	2019	NSCLC	Intertrabecular Vertebral	CEA
Zhou et al. (Zhou et al., 2017)	2017	NS	Bone	CA-125, ALP
Liu et al. (Zhang et al., 2017)	2017	Adenocarcinoma	Brain, Lymph node	CYFRA21-1
Wu et al. (Wu et al., 2017)	2017	NSCLC	Lymph node	MicroRNA-422a
Chu et al. (Chu et al., 2017)	2017	Adenocarcinoma	Lymph node	CLSTNI, CLU, NGAL
Chen et al. (Chen et al., 2015a)	2015	NSCLC	Brain	NSE
Chen et al. (Chen et al., 2015b)	2015	NS	Lymph node	CYFRA21-1, CEA
Lee et al. (Lee et al., 2012)	2012	NSCLC	Brain	CEA
Cabreraalarcon (Cabreraalarcon et al., 2011)	2011	NS	NS	CYFRA21-1
Cedres (Cedrés et al., 2011)	2011	NSCLC	Brain	CEA, CYFRA21-1, CA-125
Oshiro et al. (Oshiro et al., 2004)	2004	Adenocarcinoma	Liver	AFP
Pollan et al. (Pollan et al., 2003)	2003	NSCLC	NS	CA-125
Niklinskij (Nikliúski et al., 1992)	1992	NSCLC	Lymph node	SCC

In summary, the high expression of serum CEA and CYFRA 21-1 may be related to the occurrence of mediastinal lymph node metastasis in lung cancer patients. At the same time, assessment of the combination of CEA + CYFRA 21-1 can aid in the diagnosis of mediastinal lymph node metastasis in lung cancer patients. The positive expression of serum CEA + and CYFRA 21-1 is associated with the prognosis of patients with mediastinal lymph node metastasis.

However, there are some limitations, because of the large individual differences in some patients, and the small number of samples in this study, the statistical significance is not very significant, and the difference between the minimum and maximum values of CEA in patients is large, which leads to the standard deviation is too high, also The error and statistical difference that would cause the experiment are not significant, and the minimum and maximum values with obvious individual differences can be removed while ensuring the number of samples, thereby reducing the error.

## Data Availability

The original contributions presented in the study are included in the article/Supplementary Material, further inquiries can be directed to the corresponding author.

## Abbreviations

AFP, alpha fetoprotein; ALP, alkaline phosphatase; AUC, area under the curve; CEA, Cancer embryonic antigen; CT,Computerized tomography; CYFRA 21-1, cytokeratin fragment 19; HB, calcium hemoglobin; MRI, magnetic resonance imaging; NSE, and neuron-specific enolase; ROC, receiver operating curve; TPSA, total prostate specific antigen.
